# Impact of Heat Treatment on the Microbiological Quality of Frass Originating from Black Soldier Fly Larvae (*Hermetia illucens*)

**DOI:** 10.3390/insects13010022

**Published:** 2021-12-24

**Authors:** Noor Van Looveren, Dries Vandeweyer, Leen Van Campenhout

**Affiliations:** 1 Research Group for Insect Production and Processing, Department of Microbial and Molecular Systems (M2S), Geel Campus, KU Leuven, Kleinhoefstraat 4, 2440 Geel, Belgium; noor.vanlooveren@kuleuven.be (N.V.L.); dries.vandeweyer@kuleuven.be (D.V.); 2 Leuven Food Science and Nutrition Research Centre (LFoRCe), KU Leuven, Kasteelpark Arenberg 20, 2463, 3001 Leuven, Belgium

**Keywords:** black soldier fly larvae, *Hermetia illucens*, insect frass, heat treatment, foodborne pathogens

## Abstract

**Simple Summary:**

Industrially produced insects can convert organic waste streams into proteins and other valuable products. The black soldier fly (*Hermetia illucens*) belongs to the most promising insect species. The residue, called frass, which remains after the production of the larvae, shows great potential as a soil improver and plant fertilizer, but it also contains a large amount of microorganisms, potentially including pathogenic species for humans. To minimize safety risks upon consumption of crops fertilized by this frass, a treatment that reduces pathogenic organisms, such as heating, is required. In this study, the impact of a heat treatment of 70 °C for 60 min (as prescribed by legislation) on frass of black soldier fly larvae was evaluated. The treatment resulted in a small reduction of the total microbial counts, and bacterial endospores were not at all reduced. However, when the foodborne pathogens *Salmonella* and *Clostridium perfringens* were added to the frass, the heat treatment led to undetectable amounts for both pathogens, as well as for Enterobacteriaceae. Consequently, a heat treatment of 70 °C for 60 min is likely appropriate to meet the microbiological criteria for the application of insect frass as biofertilizer or soil improver.

**Abstract:**

Since black soldier fly larvae (BSFL, *Hermetia illucens*) are being produced at substantial volumes, concomitantly large amounts of the resulting by-product, called frass, are generated. This frass can potentially be applied as valuable plant fertilizer or soil improver. Since frass carries high microbial counts, potentially including foodborne pathogens, safety problems for consumers should be prevented. A heat treatment of 70 °C for 60 min is proposed to reduce harmful organisms in insect frass, based on EU regulations ((EU) No. 2021/1925). This study evaluated for the first time the impact of the proposed heat treatment on BSFL frass. This was done by applying the treatment on uninoculated frass as well as on frass inoculated with *Salmonella* or *Clostridium perfringens* at 5.0 log cfu/g. The heat treatment resulted in a reduction (maximum one log-cycle) of total viable counts and did not noticeably reduce bacterial endospores. In contrast, Enterobacteriaceae counts were reduced to below the detection limit (10 cfu/g). Heat treatment of inoculated frass resulted in absence of *Salmonella* in 25 g of frass and reduction of vegetative *C. perfringens* to below the detection limit (1 cfu/g). The proposed heat treatment appears to be appropriate to meet the microbiological regulations for insect frass.

## 1. Introduction

Interest in the production of insects as valuable protein source for food and feed purposes and for other purposes is increasing. One of the main reasons is their potential to convert a broad range of low-value organic waste streams into high-value biomass at high bioconversion rates [1,2]. The black soldier fly (*Hermetia illucens* L., Diptera: Stratiomyidae) has become one of the most promising insect species, which is reflected in rearing of the larvae by numerous companies [3]. A by-product created during this mass production of black soldier fly larvae (BSFL) is the residue, or also called frass, which contains non-consumed feeding substrate, insect excrements, and (parts of) dead insects [4,5,6,7]. The increase in production volumes of BSFL will inevitably lead to substantial amounts of frass [6]. The frass shows a great potential to be applied as a valuable plant biofertilizer (improving the nutrient content of the soil, and hence having a direct effect on plant growth) and/or as soil improver (improving other (physical) properties of the soil such as structure, and hence having an indirect effect on plant growth) [4,5,7]. The application of frass can be considered as a step towards more sustainable agriculture and a circular economy [8]. Several studies [9,10,11,12] reported comparable or even better plant characteristics, such as crop growth, yield, and nutritional quality, when BSFL frass was applied than when other, commercial organic and inorganic fertilizers were applied.

The microorganisms present in organic plant fertilizers can play multiple roles [8]. They are known to have a positive effect on plant growth, on tolerance to abiotic stress, and on resistance to pathogens and pests [8,13]. Conversely, when applied in the soil, frass can also transmit plant pathogens [14] and human pathogens [15]. Hence, using insect frass as fertilizer and/or soil improver for cultivating crops can introduce safety risks for consumers. Microbiological risk assessment should therefore be thoroughly conducted [16].

Processed animal manure for fertilizer and soil improver purposes is subjected to quality control as defined by European Regulations (Regulation (EC) No. 1069/2009 and (EU) No. 142/2011) [17,18]. These regulations, however, are not specifically formulated for insect frass. In this context, a new Regulation (EU) No. 2021/1925 implementing Regulation (EU) No. 142/2011 relating to the requirements for placing on the market of certain insect products and the adaptation of a containment method was recently published and adopted [19]. The regulation suggests aligning the standards of the EU market of insect frass with the requirements applying for processed manure. These requirements include a heat treatment of 70 °C for at least 60 min and imposes compliance with several microbiological criteria (Table 1).

The aim of this study was to examine the impact of a heat treatment of 70 °C for 60 min on the microbiological quality and safety of BSFL frass. First, a broad microbiological screening was performed on untreated frass to examine its microbiological quality. Next, BSFL frass was inoculated or not with *Salmonella* or *Clostridium perfringens* and then heat treated according to the above process parameters.

## 2. Materials and Methods

### 2.1. Black Soldier Fly Larvae Rearing Conditions and Frass Generation

Black soldier fly larvae (BSFL) were reared at a research institution for insects (Inagro, Rumbeke-Beitem, Belgium). Five-day-old larvae (20.000 specimens) were grown on 10 kg freshly prepared substrate, consisting of chicken feed (33%) and water (67%) and characterized by a pH of 6.5. A single feeding point was used and the larvae were reared on this substrate in plastic boxes of 60 cm × 40 cm covered with a lid with ventilation holes in the corners. The boxes were placed in a 24 h dark climate chamber at a temperature of 27 °C and a relative humidity of 60%. The larvae were harvested after 11 days, and the frass was collected immediately after the harvesting, shipped to the laboratory and stored at 4 °C. In total, a batch of frass of about 5 kg was obtained.

### 2.2. Experimental Set-Up, Heat Treatment and Samping

**Figure 1:**
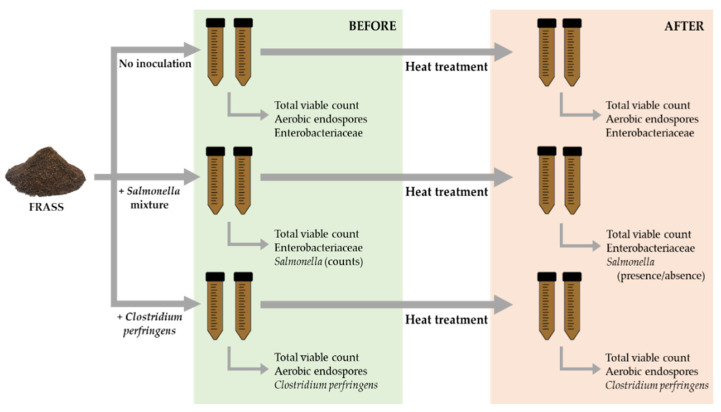
Design of one heat treatment (experiment) with three different inoculation conditions and associated analyses before and after the heat treatment. The experiment was performed three times consecutively, using other subsamples of the frass.

First, the overall microbiological quality of the fresh, untreated frass was determined on three different subsamples of the 5 kg batch. Then, three consecutive heat treatments (i.e., three heat treatments performed after each other, each on another subsample taken from the batch) were performed, further referred to as ‘experiment’. The design of one heat treatment or experiment and the associated analyses are illustrated in Figure 1. Prior to the heat treatment, one part of the frass was not inoculated, another part was inoculated with *Salmonella* and a third part with *C. perfringens* (three inoculation conditions, see also Section 2.3). Each part was homogenized using a sterile spoon. Then, for each inoculation condition, two tubes (i.e., two replicates) were completely filled with 15 g frass. The tubes were transferred into a temperature-controlled water bath (WNB14, Memmert, Schwabach, Germany), in which the samples were held at 70 °C for 60 min after reaching the treatment temperature in the coldest point of the tubes (as determined in preliminary experiments, not described here). An additional tube (not belonging to the six mentioned before) was used to monitor the temperature using a temperature data logger (Escort Junior High Temperature Logger HJ-FP-V-16-CI, Escort Messtechnik AG, Aesch, Switzerland). After the heat treatment, the tubes were transferred to an ice bath for about 30 min to cool down before analysis. Microbial counts were determined before as well as after the heat treatment.

### 2.3. Bacterial Strains and Inoculation

Three inoculation conditions were included in each experiment: no inoculation, inoculation with *Salmonella*, and inoculation with *C. perfringens*. The *Salmonella* culture consisted of a mixture of two *Salmonella enterica* strains, i.e., *Salmonella enterica* subsp. *enterica* serovar Infantis (LMG 18746) and *Salmonella enterica* subsp. *enterica* serovar Typhimurium (LMG 18732), both obtained from the Belgian Coordinated Collection of Microorganisms (BCCM, Ghent, Belgium). To avoid difficulties with overgrowth of non-specific background bacteria in the detection of *Salmonella*, kanamycin-resistant *Salmonella* strains were generated from these strains as described by De Smet et al. [20], by using the system based on a temperature sensitive pHSG415-tnsABCD helper plasmid and a modified mini-Tn7 delivery system [21]. In short, the delivery plasmid pUC18R6K-mini-Tn7T-pCS26-KmRsig70_c10 LUX was introduced via electroporation in the selected *Salmonella* strains to incorporate a kanamycin-resistance gene. The kanamycin-resistant species were cultivated overnight at 37 °C in Luria Bertani broth (10 g/L tryptone (VWR, Leuven, Belgium), 5 g/L yeast extract (VWR), 10 g/L NaCl), supplemented with kanamycin (50 µg/mL, Thermo Fisher Scientific, Merelbeke, Belgium). The *C. perfringens* strain used for inoculation (LMG 11264) was also obtained from the BCCM and was anaerobically grown overnight at 37 °C in thioglycolate broth (Biokar Diagnostics, Beauvais, France). Anaerobic conditions were created using gas generating sachets (Oxoid Anaerogen 2.5 L Sachet, Thermo Fisher Scientific) in anaerobic jars (VWR), with anaerobic indicator strips (Oxoid Resazurin Anaerobic Indicator, Thermo Fisher Scientific) to evaluate anaerobic conditions.

All individual strain cultures were diluted with peptone physiological salt solution (PPS, 0.85% NaCl, 0.1% peptone, Biokar Diagnostics) to a density of 3.0 McFarland units (DEN-1 McFarland 166 Densitometer, Grant instruments, Cambridge, UK), determined in preliminary experiments to correspond with approximately 8.0 log cfu/mL. For *Salmonella*, the two diluted strain cultures were mixed in equal volumes to obtain the final inoculation mixture. To simulate a ‘worst case’ situation in which the frass would be highly contaminated with *Salmonella* or *C. perfringens*, 100 µL of the *Salmonella* mixture was added drop by drop to 100 g of frass, or 250 µL of the *C. perfringens* inoculation suspension was added to 70 g of frass to reach a concentration for each of the inoculants of around 5.0 log cfu/g. For the uninoculated condition, 100 µL of sterile PPS was added to 100 g of frass in an identical way to the inoculated experiments. The frass was then homogenized using a sterile spoon and transferred to sterile tubes for the heat treatment as described earlier.

### 2.4. Determination of Intrinsic Parameters

Before the heat treatment, uninoculated frass was subjected to measurement of pH, water activity (a_w_) and moisture content. To measure the pH, a digital pH meter (Portamess 911, Knick, Berlin, Germany, with SI analytics electrode, Mainz, Germany) was used at room temperature, after addition of 17 mL of demineralized water to 10 g of frass according to the method of [22]. A water activity meter (LabMaster a_w_, Novasina, Lachen, Switzerland) was used to determine the water activity (i.e., the amount of water available for microbial growth), after water activity and temperature (25 °C) were stable for 5 min. Moisture content (i.e., the total amount of water present) was determined by calculating the difference in weight of 5 g frass before and after overnight oven drying at 105 °C. All measurements of pH, a_w_ and moisture content were performed in triplicate.

### 2.5. Microbiological Analyses

All microbial counts were performed according to the ISO-standards for microbial analyses of food and feed as described by [23]. For each sample, a primary dilution was obtained by diluting the frass in sterile PPS (1:10), followed by a homogenization in a stomacher (BagMixer, Interscience, Saint Nom, France) for 60 s. From this primary dilution, a tenfold dilution series was made and plated on different media. For the broad microbiological screening of the untreated, uninoculated frass, total viable counts were determined on Plate Count Agar (PCA, Biokar Diagnostics) after incubation at 30 °C for 72 h. Enterobacteriaceae were determined on Violet Red Bile Glucose agar (VRBG, Biokar Diagnostics) after incubation at 37 °C for 24 h. Presumptive Enterobacteriaceae colonies were confirmed using oxidase strips (Carl Roth, Karlsruhe, Germany), followed by inoculation of oxidase negative colonies in oxidation/fermentation (O/F) glucose medium (VWR) and incubation at 30 °C for 24 h. Colonies that were oxidase negative and also fermented glucose, observed by the development of a yellow color, were confirmed to be Enterobacteriaceae. Lactic acid bacteria (LAB) were counted on de Man Rogosa Sharpe agar (MRS, Biokar Diagnostics) after incubation at 30 °C for 72 h. Yeasts and molds were counted on Dichloran Rose Bengal Chloramphenicol agar (DRBC, Biokar Diagnostics) after incubation at 25 °C for 5 days. Iron Sulphite agar (ISA, Oxoid, Thermo Fisher Scientific) was used for counting sulphite-reducing clostridia after anaerobic incubation at 37 °C for 48 h. *Salmonella* spp. counts were determined on a chromogenic RAPID’*Salmonella* agar (Bio-Rad Laboratories, Temse, Belgium), after incubation at 37 °C for 24 h. Coagulase-positive staphylococci, determined as indicator organisms for the food pathogen *Staphylococcus aureus*, were determined on Vogel-Johnson agar (VJA, Sigma-Aldrich, Overijse, Belgium), supplemented with a 1% potassium tellurite solution (20 mL/L VJA, Sigma-Aldrich). Total *C. perfringens* counts (i.e., both vegetative cells and endospores) were determined on Tryptose Sulphite Cycloserine agar (TSC, Biokar Diagnostics), supplemented with D-cycloserine (200 mg/500 mL TSC, Biokar Diagnostics), after anaerobic incubation at 37 °C for 24 h. Presumptive *C. perfringens* colonies were subjected to a confirmation test by first resuscitating them in thioglycolate broth for 24 h at 37 °C, and then incubating them at 46 °C for 24 h in lactose sulphite broth containing a Durham tube. A confirmation test is positive if the lactose sulphite broth colors black (iron sulphide precipitate) and gas is formed in the Durham tube. Bacterial endospores were determined after giving the 10^−1^ dilution a heat shock (80 °C for 10 min), followed by preparing a tenfold dilution series and plating on PCA and incubation at 37 °C for 24 h for aerobic endospores, and plating on TSC and anaerobic incubation at 37 °C for 24 h for *C. perfringens* endospores. Presumptive *C. perfringens* endospore colonies were confirmed using the same test as for total *C. perfringens* colonies.

Depending on the inoculation condition before the heat treatment (no inoculation, inoculation with *Salmonella*, or inoculation with *C. perfringens*), different counts were determined. For the uninoculated frass, total viable counts and counts of aerobic endospores and Enterobacteriaceae were determined. For frass inoculated with *Salmonella*, total viable counts and counts of Enterobacteriaceae and *Salmonella* were determined. Before the heat treatment, *Salmonella* was counted on RAPID’*Salmonella* agar, supplemented with kanamycin (50 µg/mL agar), after incubation at 37 °C for 24 h. After the heat treatment, presence of *Salmonella* in 25 g frass was determined using the RAPID’*Salmonella* short protocol (Bio-Rad Laboratories), according to the ISO 16140 standard. For this short protocol, 25 g of heat-treated frass were first diluted in 225 mL of buffered peptone water (Bio-Rad Laboratories), supplemented with a selective RAPID’*Salmonella* capsule (Bio-Rad Laboratories) and incubated at 41.5 °C for 18 to 22 h for selective enrichment. Next, 10 µL of the enriched solution was spread plated on RAPID’*Salmonella* agar with kanamycin and incubated at 37 °C for 24 h. Presumptive colonies, found before as well as after the heat treatment, were confirmed using a *Salmonella* latex kit (Bio-Rad Laboratories). Finally, for the frass inoculated with *C. perfringens*, total viable counts and counts of aerobic endospores as well as total *C. perfringens* and *C. perfringens* endospores counts were determined. For each inoculation condition the heat treatment was repeated three times, with two replicates per heat treatment and plating was performed in duplicate to calculate the mean and standard deviations, expressed in log cfu/g.

### 2.6. Statistical Analyses

Data before and after the heat treatment for the same inoculation condition were statistically compared using an Independent-Samples *t*-test in case of normal distribution (evaluated by Shapiro-Wilk test) and equal variances (evaluated by Levene test). When equal variances could not be confirmed, a Welch’s *t*-test was used. For plate counts below the detection limit, the detection limit itself was used for statistical analysis. All statistical analyses were performed using the JMP Pro 15 software package from SAS, considering a significance level of α = 0.05.

## 3. Results

### 3.1. Intrinsic Parameters and Microbiological Counts of Untreated Frass

As intrinsic parameters, the pH, a_w_ and moisture content of fresh, untreated frass were determined. The results are shown in Table 2. A near-neutral average pH of 7.21 was obtained. While a high water activity of 0.98 was measured for the frass, the average moisture content was only 52.5%.

Various microbiological parameters were determined on the untreated frass to investigate its microbiological quality. The results of this microbiological screening are also shown in Table 2. A high average value (9.5 log cfu/g) was observed for the total viable count. Regarding the microbial subgroups, the highest average count was observed for the LAB (8.1 log cfu/g), but also the Enterobacteriaceae (7.7 log cfu/g) and aerobic endospores (6.2 log cfu/g) showed high average counts. Compared to the other microbiological parameters, yeasts and molds showed a somewhat lower average value (4.4 log cfu/g). Of the three foodborne pathogens studied (Table 2), only coagulase-positive staphylococci counts were found to be above the detection limit, and even high values (7.5 log cfu/g) were observed. No *Salmonella* colonies were observed on the RAPID’*Salmonella* plates, resulting in values below the detection limit of 2.0 log cfu/g. However, it should be noted that in this study the RAPID’*Salmonella* plates were completely overgrown by numerous other non-specific colonies (background microbiota), which could possibly conceal the presence of *Salmonella* colonies. At last, all counts for sulphite-reducing clostridia and *C. perfringens* (total counts as well as endospores) were below the detection limit of 1.0 log cfu/g in this study.

### 3.2. Microbial Counts before and after Heat Treatment of Frass

To evaluate the impact of a heat treatment of 70 °C for 60 min on the microbiological quality and safety of BSFL frass, microbial counts were determined before as well as after the heat treatment for the three different inoculation conditions. Results are shown in Table 3. Values for the total viable count were high for untreated frass, as also indicated in the overall microbiological screening (Table 2). Surprisingly, a heat treatment of 70 °C for 60 min on uninoculated frass still resulted in a high average total viable count of 8.5 log cfu/g, although the reduction was statistically significant (*p* < 0.001). Furthermore, the thermal treatment was insufficient to reduce the aerobic endospores, illustrated by equal counts before and after the heat treatment. In contrast, the Enterobacteriaceae, which were present in high amounts (7.1 cfu/g) in the untreated frass, were reduced to below the detection limit of 10 cfu/g (or 1.0 log cfu/g in Table 3) for all six replicates after the heat treatment in the not inoculated condition.

**Figure A1:**
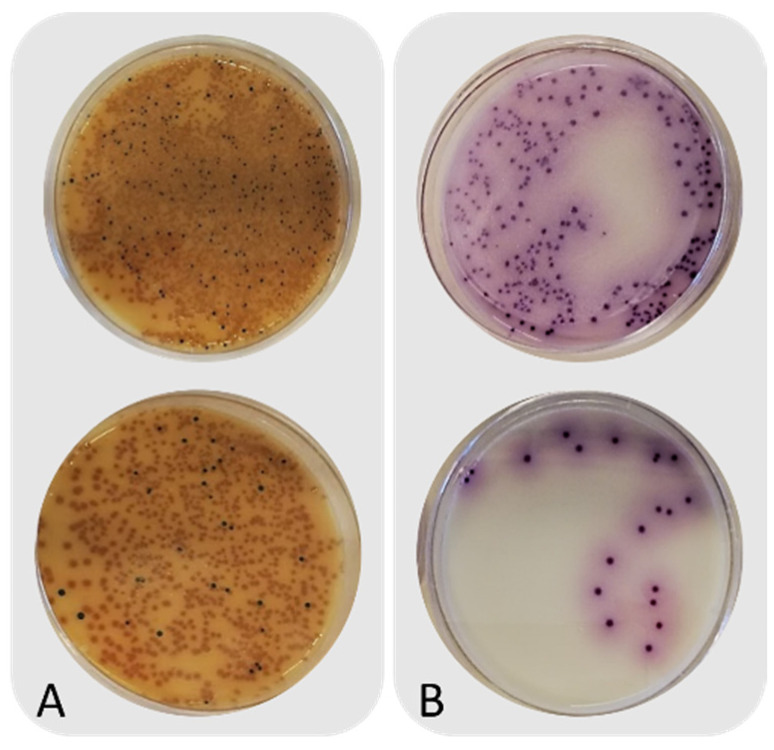
Determination of *Salmonella* spp. in inoculated frass using RAPID’*Salmonella* agar plates (**A**) without kanamycin, overgrown by background microbiota (blue and brown colonies represent other Enterobacteriaceae and *Proteus* spp. respectively), (**B**) supplemented with kanamycin and with countable kanamycin-resistant *Salmonella* colonies (purple colonies represent *Salmonella* spp.).

For inoculation with *Salmonella*, a mixture of *Salmonella* enterica strains was used. Preliminary experiments showed that accurate counting of wild-type *Salmonella* strains on the RAPID’*Salmonella* medium after inoculation in the frass was hindered by an abundant background microbiota (see also Figure A1 of Appendix A). In order to prevent this problem, genetically modified kanamycin-resistant *Salmonella* strains were used, according to [20], who reported the same problem for the rearing substrate and for BSFL samples in inoculation trials. Combination with the use of kanamycin in the medium resulted in countable *Salmonella* colonies on the RAPID’*Salmonella* medium (see also Figure A1 of Appendix A). Results for the total viable count, Enterobacteriaceae and *Salmonella* are shown in Table 3. Counts for the total viable count were similar to the counts for the uninoculated condition, and were reduced by one log-cycle (*p* < 0.001). The Enterobacteriaceae were reduced from a high average count (6.6 log cfu/g) to below the detection limit of 1.0 log cfu/g for all six replicates after the heat treatment. After inoculation of the frass, the target *Salmonella* concentration of 5.0 log cfu/g was approached with an actual value ranging from 5.1 to 5.6 log cfu/g. After the heat treatment, absence of *Salmonella* in 25 g frass was observed for all six replicates.

Since previous studies reported the occurrence of *C. perfringens* vegetative cells and endospores in BSFL [24] and BSFL frass [25,26], the effectiveness of a heat treatment of 70 °C for 60 min on the reduction of *C. perfringens* was also included in this work (Table 3). The total viable count was only slightly reduced from 9.0 log cfu/g to 8.3 log cfu/g (*p* = 0.002), comparable to the other inoculation conditions. Only a very small (yet statistically significant; *p* < 0.001) reduction of the aerobic endospores from 5.5 log cfu/g to 5.1 log cfu/g was observed. After inoculating the frass with *C. perfringens*, an inoculation level close to the target concentration of 5.0 log cfu/g was reached for total *C. perfringens*, being 4.7 to 5.0 log cfu/g. All presumptive colonies for the inoculum and for the inoculated frass before the heat treatment were confirmed to be *C. perfringens*. In contrast, none of the colonies on the TSC plates for counting *C. perfringens* endospores before the heat treatment were confirmed to be *C. perfringens*. This indicates that the inoculum contained only vegetative *C. perfringens* cells. After the heat treatment, colonies were still present on the TSC plates, but none of them were confirmed as *C. perfringens*, implying that vegetative *C. perfringens* cells were reduced to below the detection limit of 1 cfu/g.

## 4. Discussion

### 4.1. Microbiological Quality of Untreated Frass

When evaluating the intrinsic parameters of the untreated BSFL frass (Table 2), the pH of 7.21 was lower than reported for industrial large-scale processes in other studies [25,26]. The total amount of water present (represented by the moisture content) was moderate, but most of it present was available for microorganisms (represented by the a_w_).

Data from Table 2 indicate high microbial counts for fresh, untreated frass. Only a few other studies investigated similar microbiological parameters on BSFL frass [25,26,27,28]. The rearing conditions used in these studies and obtained microbial counts are given in Supplementary Table S1. Differences in rearing conditions, for example the sampling moment in the rearing cycle, feed rates, and larval density, can differ between studies and can influence the microbiological counts of the frass [26,27]. In addition, differences in feeding system (single or multiple feeding point) could influence the intrinsic parameters of the substrate/frass and consequently the microbial numbers and/or composition in the substrate. Multiple feeding systems possibly render fresher frass with a higher moisture content and a lower pH. Moisture content (or even better water activity) and pH belong to the most important parameters affecting microbial growth. Yet, research is needed to determine the exact influence of the feeding system on the microbiota of frass.

The values shown in Table 2 were comparable to data for frass reported by [26]. These authors studied several BSFL rearing cycles at laboratory scale and in large scale rearing facilities at different locations, each using other substrates and/or rearing conditions. They reported total viable counts for frass ranging from 8.5 to 10.2 log cfu/g over all rearing cycles. Counts for the Enterobacteriaceae ranged from <5.0 to 9.5 log cfu/g and for the LAB from <5.0 to 9.8 log cfu/g. In another study [27], the frass of BSFL was investigated after a 12-day rearing cycle on canteen food waste and household food waste. Total viable counts of 8.6 to 10.0 log cfu/g were reported, next to counts of 7.5 to 8.1 log cfu/g for LAB. Yet another study [28] reported similar values as in our study for frass of BSFL reared until prepupal stage on coffee silverskin with or without microalgae. They reported average values of 9.3 to 9.8 log cfu/g and 7.6 to 8.2 log cfu/g for the total viable count and LAB, respectively. For the Enterobacteriaceae, however, lower average counts of 3.7 to 5.0 log cfu/g were observed. A further study [25] showed an average total viable count of 8.7 log cfu/g for frass of BSFL reared on a mixture of potato starch, a wheat-and-potato-processing-product and protein kibbles.

Table 2 also reveals high values for aerobic endospores in the frass. Again, these values are in line with data of other studies [25,26,28], in which average aerobic endospore counts were reported ranging from 4.2 to 7.6 log cfu/g. In contrast, counts of yeasts and molds were somewhat lower than for the previous microbial parameters (Table 2). This is not unexpected, since yeasts and molds represent a small fraction of the (gut) microbiome of BSFL [24,26]. Another study [26] indicated a broad range of 3.6 to 6.3 log cfu/g for fungi in the frass of industrially reared BSFL, whereas other studies [27,28] reported higher average values for yeasts and molds.

The presence of high amounts of coagulase-positive staphylococci in the BSFL frass was not surprising, since this bacterial subgroup was also counted in large amounts (7.0 to 7.3 log cfu/g) in a previous study [28]. Furthermore, the presence of *C. perfringens* in BSFL frass was reported by other studies [25,26], although only low average counts of 2.2 log cfu/g were reported, of which the largest part consisted of vegetative cells. The absence of *Salmonella* in BSFL frass was previously indicated in literature [28].

The large microbial load in BSFL frass may contain microorganisms that promote plant growth (Plant Growth Promoting Microorganisms or PGPMs). Some reports indicate a beneficial influence of insect residue on abiotic and biotic stress resistance in plants, due to the presence of specific microorganisms, such as several species of the genera *Bacillus* and *Klebsiella*. PGPMs have been shown to enhance the health of plants by facilitating the absorption of nutrients or inhibiting the growth of plant pathogens [29,30].

### 4.2. Impact of Heat Treatment on Microbiological Quality of Frass

To the best of our knowledge, there are no data available for insect frass that evaluate the effect of the proposed heat treatment of 70 °C for 60 min to meet the required criteria of Table 1. In this study, the impact of this heat treatment on BSFL frass was evaluated using three different inoculation conditions.

For uninoculated frass, the heat treatment at 70 °C for 60 min slightly reduced the total viable counts, but there was no effect at all on the aerobic endospore count. The treatment did not have a major impact on the total viable counts and a higher reduction was expected. This cannot completely be explained by the aerobic endospore counts which were (almost) not affected by the treatment. In addition to that, it must be kept in mind that when applying heat treatments to a certain matrix (food, feed, frass or whatever matrix), depending on its composition, the matrix may have a protective effect (e.g., due to a high lipid content) to the microorganisms it contains. Different types of frass may therefore protect microorganisms to a different extent, which is why total viable counts may not (always) be reduced significantly and results obtained for one frass type cannot simply be extrapolated to another. The fact that the total viable counts were reduced only slightly may be promising, in that it may mean that potential PGPMs present in the frass can survive the heat treatment. More research on the presence of PGPMs and on their survival of decontamination strategies for frass is needed, however.

In general, bacterial endospores are considered to be heat resistant [31,32], because they contain almost no water and because of their composition [33]. Inactivation of bacterial endospores mostly requires a temperature of 30 to 40 °C higher than inactivation of vegetative cells of the same strain [34,35]. Based on values for the decimal reduction time (D-value, time to reduce the counts by one log cycle at a certain temperature and in a certain matrix) published for the spores of a number of bacterial species [36,37], only a very small reduction could have been expected. For example, reported D-values are about 30 min for *B. cereus* and *C. perfringens* endospores at 85 °C and 90 °C, respectively, [38] and 7–13 min for *C. botulinum* E at 74 °C [36]. However, several factors affect the impact of a heat treatment on microbial inactivation (including inactivation of spores), such as the water activity, the water mobility, the composition and the physical structure of the matrix [36], and these factors also interfere with each other’s effect. Frass may have a protective effect on bacterial spores, but more research is needed here. The fact that bacterial endospores are still present in the heat-treated frass is not necessarily a problem, because no information on the composition of this microbial group (i.e., which species, pathogenic or not, are present) is available for this study. Yet, a point of attention for bacterial spores is that when they are not killed by heat processing, they may not only survive the treatment but also be activated, i.e., be triggered to germinate and form vegetative cells. In future research, it may therefore be worthwhile to store heat treated frass and monitor microbial counts during storage. In addition, it might be interesting to investigate in the future whether a short pre-heat treatment step of the frass can activate the endospores before the actual heat treatment of 70 °C for 60 min (a process which is also known as tyndallization [39]), which would then also kill the activated endospores.

Unlike the total viable count and the aerobic endospores, the Enterobacteriaceae counts were highly reduced. Although no specific measurements were performed for *E. coli*, as imposed by Regulation (EU) No. 142/2011 (Table 1), it can be deduced from our results that all tested samples were below 1000 cfu/g after processing, since *E. coli* is a member of the Enterobacteriaceae family and would also grow on the VRBG agar used [40]. The inactivation of the Enterobacteriaceae is not surprising, since they are generally heat sensitive [41]. Likewise, another study [42] reported a reduction of *E. coli* in poultry litter from 8.8 log cfu/g to undetected levels after about 45 min at 60 °C.

In the frass inoculated with *Salmonella*, the treatment resulted in the absence of the pathogen in 25 g frass in all replicates, in line with the legal criterium. This temperature-time combination can be expected to be sufficient to obtain a 5-log reduction of *Salmonella*, based on a review [43] reporting an average D-value of 0.2 min at 70 °C for *Salmonella* spp. in various food products. This means that a heat treatment of 0.2 min at 70 °C is (on average) required for a tenfold reduction (=1 log-reduction) of *Salmonella* spp. Nevertheless, there are several factors that influence the heat resistance of a pathogen, such as the surrounding matrix (as for spores), strain variations, the growth phase of the culture and the experimental conditions [44]. While to the authors’ knowledge no other studies on thermal inactivation of pathogens in insect frass are available, various studies were performed with other animal manure types, and mostly chicken litter. One study [42] examined the survival of *Salmonella* Typhimurium in a chicken litter slurry with an initial concentration of 8.6 log cfu/g, and reported that *Salmonella* was not detected anymore after about 45 min at 60 °C. In contrast, other studies [45,46] reported that a heat treatment of 70 °C for 60 min was not sufficient for the required reduction of *Salmonella*. For example, a first study [45] reported the survival of *Salmonella* spp. after a treatment of aged chicken litter with 20% moisture content at 70 °C for about 1.5 to 2 h. Another study [46] also detected *Salmonella* in fresh chicken litter with a moisture content of 30% and 40 to 50%, after heat treatments of 105 and 90 min at 70 °C, respectively. For 6 months old chicken litter, *Salmonella* was even still detected after a treatment of more than 300 min at 70 °C [46]. In conclusion, the moisture content and age of the faeces seemed to have an important influence on the effectiveness of the heat treatment of chicken litter, next to the processing temperature and time. Further research is necessary to investigate whether this is also applicable for insect frass. Although the standards of (EU) No. 142/2011 apply to samples taken ‘during or on withdrawal from storage’ for evaluation of *Salmonella* (Table 1), the storage of heat-treated frass was not included in the scope of this study. However, based on our results, it can be assumed that if no *Salmonella* was present immediately after the heat treatment, there will be no growth during storage of the frass. The protocol used included a selective enrichment step (see Section 2) and therefore, if sub-lethally injured *Salmonella* cells would still be present (and potentially grow later during storage of the frass), they would be detected in the analysis. Nevertheless, future research on stored heat-treated frass can be recommended and should confirm that if *Salmonella* is not present immediately after treatment, it does not redevelop during storage.

In the frass inoculated with *C. perfringens* vegetative cells, a heat treatment of 70 °C for 60 min seemed to be sufficient to reduce the vegetative cells to below the detection limit of 1 cfu/g. Indeed, vegetative *C. perfringens* cells have a D-value of 0.2 to 0.4 min at 70 °C [38,43], which implies that a 5-log reduction should be already achieved after about 1 to 2 min at 70 °C. The conclusion could have been different if also *C. perfringens* endospores had been inoculated in the frass, since bacterial endospores are more heat resistant than vegetative cells. Based on the data of previous studies [38,43], more than 50 to 100 h at 70 °C would be necessary for only one log reduction of *C. perfringens* endospores. To inactivate bacterial endospores, a sterilization temperature of at least 120 °C for 3 min is required [43]. However, such a time-temperature combinations are likely to have a detrimental impact on the chemical quality of the frass and application opportunities for plant growth.

**Figure A2:**
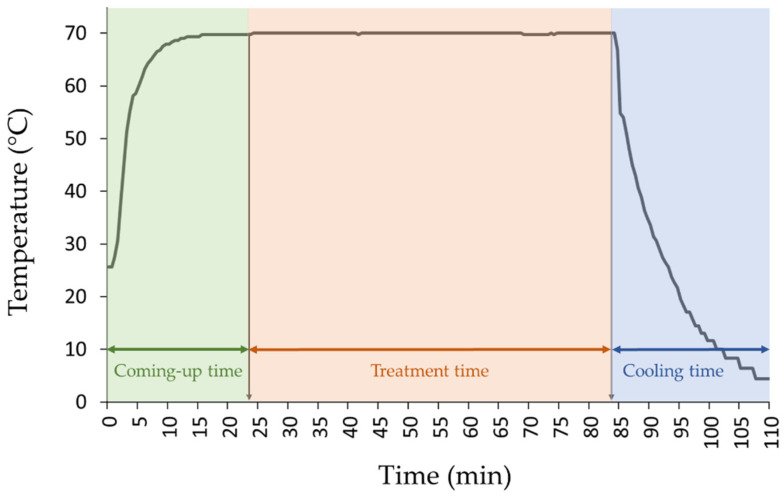
Temperature profile (coming-up phase, treatment phase and cooling phase) of the heat treatment (70 °C for 60 min) applied on the BSFL frass samples.

It is plausible that the coming-up phase of the heat treatment, i.e., the period in which the target temperature of 70 °C is reached in the coldest point of the sample, also had a reducing effect on the microbial counts, in addition to the reducing effect of the holding phase (60 min at 70 °C). For this experiment, the coming-up time was 24 min. The complete temperature profile is shown in Appendix A (Figure A2). A previous study [42] calculated the reduction of *E. coli* and *Salmonella* during the coming-up phase of a heat treatment of chicken litter. Upon reaching the target treatment temperature of 60 °C after 30 min, *E. coli* and *Salmonella* counts had already been reduced by 1.5 and 3.2 log cycles, respectively. When prescribing treatments in terms of time-temperature combinations for the holding phase, the killing effect during the coming-up phase is neglected. The holding phase can depend on the practical implementation of the heat treatment: it can differ depending on the equipment used to perform the treatment and the volume of the batch treated. However, the killing effect during the coming-up phase can be taken into account by prescribing treatments in terms of process values (or F-values), as is done for thermal processing of food [36].

While the present study provides the first evidence for the suitability of the time-temperature combination imposed by the EU legislation, the topic needs further exploration. The results obtained in this study should be confirmed using other batches of frass, preferably originating from a range of (industrial) suppliers (with evaluation of the frass immediately after the heat treatment as well as after storage, as described above). The composition of frass can obviously differ between suppliers, as they likely use different substrates, feeding regimes, hygiene measures and/or other rearing conditions. Therefore, the initial microbial load before the heat processing will differ, and hence also the surviving microbiota. In addition, the chemical composition may affect the result of the treatment, since the contents of moisture and several types of components (such as carbohydrates, fats, and proteins) determine the extent to which vegetative cells and spores are protected for the heat. Furthermore, in this work we inoculated the frass with the pathogens to be investigated, in order to be able to monitor their reduction. However, it cannot be excluded that the heat sensitivity of pathogens that are inoculated differs from the sensitivity of cells that are naturally present in the matrix. In this study, the pathogen cultures were prepared for inoculation by culturing them overnight. It was not assessed, yet it can be assumed that at the time of inoculation, the cultures were in the stationary phase. Cells naturally present in frass, for instance due to excretion by the larvae, may also be present in the exponential growth phase. It is known that cells in the latter stage are more vulnerable to stress factors such as heat, as described for instance by [47,48], than cells in the stationary phase. On the other hand, the *Salmonella* strains used in our study were transformed to be kanamycin-resistant, but this manipulation may have influenced their heat sensitivity. Wild-type *Salmonella* that may be present in frass may be (slightly) more resistant [49]. In addition, variation in resistance may occur between (wild-type) bacterial strains of the same genus [50]. For *Salmonella*, this was covered to some extent in this study by using a cocktail of strains for inoculation, a practice which is common in challenge tests in the food industry. Nevertheless, confirmation of the results found in this study with other bacterial strains, preferably isolated from BSFL rearing, would be useful to build more evidence for the suitability of the imposed treatment.

In the insect industry, operators aim for processing technologies with an energy input as low as possible. Therefore, the question may rise whether a less stringent time-temperature combination (lower time and/or lower temperature) or non-thermal decontamination technologies (for example radiation such as low energy electron beam, (pulsed) UV-light or pulsed electric fields) would also provide the required microbial reduction. This is also relevant for future research, when a solid insight on the currently imposed treatment is obtained. The current treatment can then be considered as a benchmark when evaluating milder treatments. Milder, tailor-made treatments could be allowed from a legislative point of view if it can be proven that they provide sufficient reduction. This study focused on the microbiological aspects of frass treatment, but next to microbiological results, data on the impact of treatments on the chemical quality of frass and ultimately on the effect of frass on plants are also necessary.

## 5. Conclusions

The uninoculated frass investigated in this study contained a high microbial load, but *Salmonella* spp. and *Clostridium perfringens* were both below their respective detection limits of 2.0 and 1.0 log cfu/g. The heat treatment imposed by the EU only slightly reduced the total viable counts by a maximum of one log-cycle and did not noticeably reduce the bacterial endospores. However, for the frass investigated, the process was sufficient to: (i) reduce high Enterobacteriaceae counts to below the detection limit of 10 cfu/g, (ii) result in absence of *Salmonella* in 25 g of frass which was inoculated with approximately 5.0 log cfu/g, and (iii) reduce vegetative *C. perfringens* to below the detection limit of 1 cfu/g in frass inoculated with approximately 5.0 log cfu/g. While more research is needed, this study demonstrated that the proposed heat treatment for BSFL frass is likely sufficient to meet the legislative microbiological regulations for insect frass for the application as plant fertilizer or soil improver.

## Figures and Tables

**Table 1:** Microbiological standards applying to the placing on the EU market of processed (digestion into biogas or composting) manure (Regulation (EU) No. 142/2011) [18].

Moment of Sampling	Microorganism	*n* ^a^	c ^b^	m ^c^ (cfu/g)	M ^d^ (cfu/g)
During or immediately after processing	*Escherichia coli*	5	5	0	1000
	or				
	Enterococcaceae	5	5	0	1000
During or on withdrawal from storage	*Salmonella*	5	0	Absence in 25 g	

^a^ number of tested samples; ^b^ number of samples of which the bacterial count may be between m and M; ^c^ threshold value (i.e., lower limit) for the number of bacteria in the sample; ^d^ maximum value (i.e., upper limit) for the number of bacteria in the sample. If one sample exceeds M, the sampled batch is considered as insufficiently processed and regarded as unprocessed manure.

**Table 2:** Intrinsic parameters and microbial counts of untreated frass of black soldier fly larvae. Results are presented as the mean of three replicates ± standard deviation.

Intrinsic Parameters	
pH (-)	7.21 ± 0.45
a_w_ (-)	0.98 ± 0.01
Moisture content (%)	52.5 ± 0.4
**Microbial counts (log cfu/g)**	
Total viable count	9.5 ± 0.1
Enterobacteriaceae	7.7 ± 1.0
Lactic acid bacteria	8.1 ± 0.1
Aerobic endospores	6.2 ± 1.2
Yeasts and molds	4.4 ± 0.3
Sulphite-reducing clostridia	<1.0
Total *Clostridium perfringens*	<1.0
*Clostridium perfringens* endospores	<1.0
*Salmonella* spp.	<2.0
Coagulase-positive staphylococci	7.5 ± 0.0

**Table 3:** Microbial counts for uninoculated and inoculated frass samples before and after heat treatment. Results are presented as the mean of three experiments x two replicates per experiment (*n* = 6) ± standard deviation.

Inoculation Condition	Sample	Microbial Counts (log cfu/g)					
		Total Viable Count	Aerobic Endospores	Entero-Bacteriaceae	*Salmonella* spp.	Total *Clostridium perfringens*	*Clostridium perfringens* Endospores
Not inoculated	Before heat treatment	9.2 ± 0.2 ^b^	5.3 ± 0.0 ^a^	7.1 ± 0.6 ^b^	n.d.	n.d.	n.d.
	After heat treatment	8.5 ± 0.3 ^a^	5.3 ± 0.3 ^a^	<1.0 ^a^	n.d.	n.d.	n.d.
Inoculated with *Salmonella*	Before heat treatment	9.3 ± 0.1 ^b^	n.d.	6.6 ± 0.4 ^b^	5.3 ± 0.2 ^b^	n.d.	n.d.
	After heat treatment	8.3 ± 0.1 ^a^	n.d.	<1.0 ^a^	Absent in 25 g ^a^	n.d.	n.d.
Inoculated with *Clostridium perfringens*	Before heat treatment	9.0 ± 0.3 ^b^	5.5 ± 0.1 ^b^	n.d.	n.d.	4.9 ± 0.1 ^b^	<1.0 ^a^
	After heat treatment	8.3 ± 0.1 ^a^	5.1 ± 0.1 ^a^	n.d.	n.d.	<1 cfu/g ^a^	<1 cfu/g ^a^

n.d. = not determined; ^a,b^ Means of samples before and after the heat treatment with the same letter in superscript within an inoculation condition and within a column do not differ significantly (*p* ≥ 0.05).

## Data Availability

Data can be found within the article and the appendices.

## Supplementary Materials

The following are available online at https://www.mdpi.com/article/10.3390/insects13010022/s1, Table S1: Microbial counts reported for BSFL frass and corresponding rearing conditions.
